# Sexual and individual foraging segregation in Gentoo penguins *Pygoscelis papua* from the Southern Ocean during an abnormal winter

**DOI:** 10.1371/journal.pone.0174850

**Published:** 2017-03-31

**Authors:** José C. Xavier, Philip N. Trathan, Filipe R. Ceia, Geraint A. Tarling, Stacey Adlard, Derren Fox, Ewan W. J. Edwards, Rui P. Vieira, Renata Medeiros, Claude De Broyer, Yves Cherel

**Affiliations:** 1 Marine and Environmental Sciences Centre, Department of Life Sciences, University of Coimbra, Coimbra, Portugal; 2 British Antarctic Survey, Natural Environment Research Council, High Cross, Madingley Road, Cambridge, UK; 3 Cardiff University, Cardiff School of Biosciences, Sir Martin Evans Building, Cardiff, United Kingdom; 4 Royal Belgian Institute of Natural Sciences, OD Taxonomy and Phylogeny, Rue Vautier 29, Bruxelles, Belgium; 5 Centre d´Etudes Biologiques de Chizé, UPR 7372 du CNRS-Université de La Rochelle, Villiers-en-Bois, France; Evergreen State College, UNITED STATES

## Abstract

Knowledge about sexual segregation and gender-specific, or indeed individual specialization, in marine organisms has improved considerably in the past decade. In this context, we tested the “Intersexual Competition Hypothesis” for penguins by investigating the feeding ecology of Gentoo penguins during their austral winter non-breeding season. We considered this during unusual environmental conditions (i.e. the year 2009 had observations of high sea surface and air temperatures) in comparison with the long term average at Bird Island, South Georgia. Through conventional (i.e. stomach contents) and stable isotopic values from red blood cells, plasma and feathers of both male and female Gentoo penguins, we showed that there were significant differences between sexes, with males feeding mainly on fish (54% by mass) followed by crustaceans (38%) whereas females fed mainly on crustaceans (89% by mass) followed by fish (4%). *Themisto gaudichaudii w*as the most important crustacean prey for males (64% by mass; 82% by number; 53% by frequency of occurrence) and females (63% by mass; 77% by number; 89% by frequency of occurrence), contrasting with all previous studies that found Antarctic krill *Euphausia superba* were generally the main prey. Stable isotopic data showed that, in terms of habitat use (based on δ ^13^C), there were significant differences in short-term carbon signatures between males and females (based on plasma and red blood cells), suggesting that both sexes explored different habitats, with females exploring more offshore pelagic waters and males feeding more in coastal benthic waters. Based on δ ^15^N, males fed on significantly higher trophic level than females (based on plasma and red blood cells), in agreement with our diet results., Thus, Gentoo penguins behave in a similar manner to other non-breeding penguins species (e.g. king, macaroni and rockhopper penguins), albeit at a smaller spatial scale (as they do not disperse as these other penguins do), in that they have a wider habitat and trophic niche during the Antarctic Winter (in comparison to Summer). We also detected individual specialization in feeding/trophic levels for each gender, with certain males feeding mainly on fish and certain females mainly on crustaceans, which may be driven the prevailing environmental conditions that lead individuals to search for alternative prey, and cause sexual diet segregation. Our results provide further information to help improve understanding about sexual segregation and individual specialization of marine organisms, while contributing valuable information on the winter diet for Antarctic monitoring programs and for modelling Antarctic marine food webs.

## Introduction

Understanding the natural variability of a marine ecosystem, and how organisms are able to adapt/acclimatize to environmental change, is crucial to the conservation and management of marine ecosystems. In the Southern Ocean, the network of food web interactions is now recognised as being important in determining the resilience, and hence response, of marine ecosystems to change [1, 2]. With the Southern Ocean currently showing signs of unusually rapid warming [3–7], habitat modification is affecting species at all trophic levels, but particularly those species at higher trophic levels that might integrate and/or amplify effects of change, e.g. penguins [8–14].

Penguins (Spheniscidae) are a major component of the Antarctic marine ecosystem, constituting approximately 80% of the avian biomass, and as such occupy an important role as meso-predators [15, 16]. Information on the diet and feeding ecology of penguins is vital for parameterising consumption models in Antarctic food webs [17–19]. However, it is currently unknown how penguin behaviour could adapt to change, over the longer term (i.e. decades), to exploit alternative prey types [17]. Some studies have suggested that penguins may have differing success in adapting to the loss of Antarctic krill *Euphausia superba* [20] or fish [21] in their diets. Such changes could then be linked to changes in populations; for example, Adélie penguins *Pygoscelis adeliae* on the Antarctic Peninsula are declining, whereas Gentoo penguins *Pygoscelis papua* [22, 23] are increasing, though data are not yet sufficiently comprehensive.

**Fig 1:**
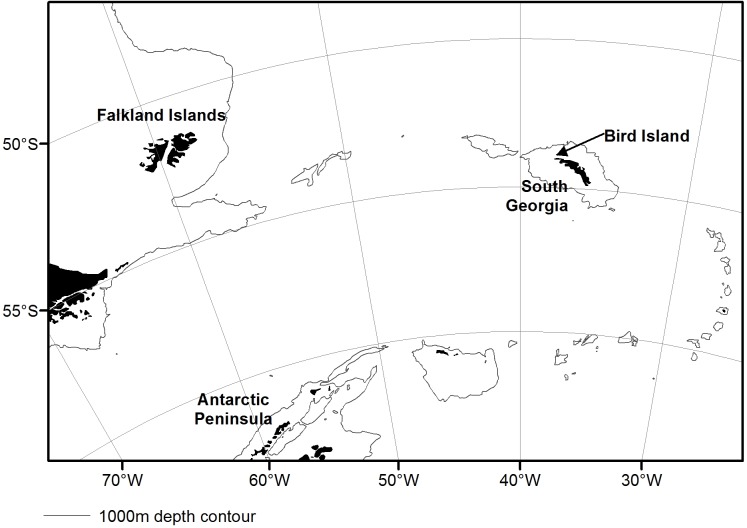
Map of the Southern Ocean, with emphasis to Bird Island, South Georgia (our study area) and the 1,000 m isobaths.

In this study, we assess the feeding ecology of a predator of the Southern Ocean, the Gentoo penguin, at Bird Island, South Georgia (54° S, 38° W; Fig 1), during the austral winter, to assess their levels of sexual and individual segregation. We consider this under unusual and extreme environmental conditions, assuming that this will ensure differences between genders are more extreme. Gentoo penguins are an inshore-feeding species and a year-round resident [24, 25], making them an excellent biological sampler of local prey abundance/availability, particularly in inshore areas. The foraging range of Gentoo penguins is generally within 30 km of South Georgia [25, 26], and their diet comprises largely Antarctic krill and fish [26–28].

Male Gentoo penguins are typically larger than females (i.e. dimorphic), although the differences can be difficult to detect visually in the field [28, 29] as body size and morphology are highly variable [30, 31]. Sexual segregation in birds is often linked to differing energetic constraints or nutrient requirements (particularly during the breeding season or related to reproduction duties) [32–34] and relatively little is known about sexual differences in the feeding ecology of seabird species during their non-breeding period [27, 28, 32, 35–38]. Furthermore, only a very few studies are available that explore individual specialization in the foraging and feeding strategies of penguins [39], with only one on individual specialization in the diet of Gentoo penguins at the Kerguelen archipelago during the austral summer [40]. Differences between individuals may have a strong impact on ecological processes (e.g. competition within/between sexes) and on population/species dynamics, as it may promote speciation [41]. Therefore, it is important to understand both diet segregation and individual specialization within a population. In our study, we looked at these mechanisms when animals have no reproductive obligation (to assess their diet flexibility) during the austral winter at Bird Island, South Georgia.

We focused on a period of unusual environmental conditions as such conditions are most likely to reveal individual differences, and because information on such conditions is important as they are likely to affect the ecology, management and conservation of Antarctic ecosystems [42].

**Fig 2:**
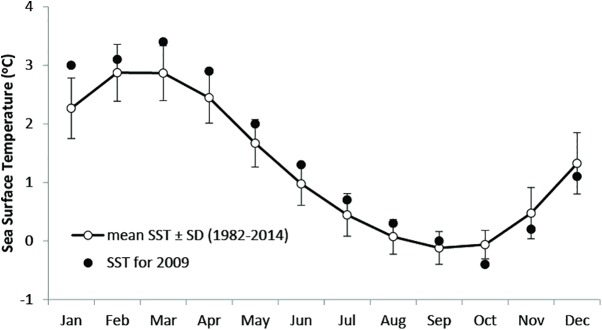
Abnormal sea surface temperatures around South Georgia in 1999 (Sea surface temperature (SST) data ± SD). Mean monthly sea surface temperature (SST) data for the 1° by 1° grid square centred at 34.5°W, 54.5°S, from the “OI.V2 Monthly SST” data set [109].

Ocean warming has been recorded at South Georgia during the austral winter (i.e. August), with a mean increase of ~2.3° C since 1925 [43]. In 2009 (when our study took place), high sea surface and temperatures occurred across many consecutive months, and coincided with extremely low catches in local fisheries and poor breeding success in higher predators at South Georgia [44, 45] (Fig 2). Prior to the fieldwork at Bird Island, a research cruise (March-April 2009) in the Scotia Sea (including around South Georgia), showed that Antarctic krill density was lower (2.2 g m^-2^) than in previous years (Antarctic krill density assessments made annually since 1996) [46]. These findings suggest that this region, during the austral winter of 2009, was atypical for prey availability/abundance to penguins and other predators in the region. Indeed, anomalous oceanographic, sea-ice and/or weather conditions can cause changes in prey availability [12, 47, 48] and are known to have negative impacts on populations of seabirds, including mass mortalities (also known as “wrecks” of seabirds) [49–52].

In the context of low Antarctic krill abundance, the behaviour of male and female penguins could provide valuable data on diet sexual segregation and individual specialization. The “Intersexual Competition Hypothesis” (see also [53] for intraspecific competition) predicts that, in dimorphic species, reduction in competition for food between sexes results from the capture of different prey types by each sex and that this is one of the main selective forces maintaining sexual dimorphism [54, 55]. Here, we had the unique opportunity to investigate the feeding ecology of Gentoo penguins during an oceanographically abnormal non-breeding season. The objectives of the study were:

- Characterize the winter diet of Gentoo penguins using conventional techniques (i.e. stomach contents), and stable isotope analyses of red blood cells, plasma and feathers to evaluate diet changes, under known environmental conditions;
- Evaluate differences in diets according to sex and individual specialization;
- Assess implications of these results on the conservation of Gentoo penguins, under extreme and low food availability.

## Material and methods

### Fieldwork and stomach content analyses

Fieldwork was carried out during the austral winter of 2009, between June and September, at Bird Island, South Georgia. The British Antarctic Survey (BAS) provided all the support related to the permits for the fieldwork (as fieldwork was carried out from a British Antarctic research base): "The animal procedures used in this this study were reviewed and approved by the Joint BAS–Cambridge University Animal Welfare and Ethical Review Committee. Permits to operate were issued by the Government of South Georgia and the South Sandwich Islands". Stomach samples were obtained each month (N = 13–15 penguins handled, always ensuring that at least 10 samples contained food) from non-breeding Gentoo penguins at one colony (known locally as Landing Beach).

For each month, all samples were collected within a 4-day period. Penguins were selected randomly when returning to the colony at dusk. Birds may, or may not have used the site for breeding, as Gentoo penguins move around the archipelago during the winter [25]. Each penguin was handled as follows: after putting a cover on the penguin’s head (to reduce stress), the bill was measured (length and depth), followed by the height of the penguin and its weight (a harness was produced to specifically hold each penguin comfortably) using a 5 kg Pesola® spring balance (Pesola AG, Barr, Switzerland). Post-molt feathers (6–8 chest feathers) and blood were collected, following Ceia *et al*. [56], adapted to penguins; the blood samples were collected using 1 ml syringes (25 G needles) and were separated into plasma and red blood cells (RBC) using a centrifuge (15 min at 3000 rpm), stored frozen (– 20°C), and later freeze-dried and homogenized prior to stable isotopic analyses. Finally, a food sample was obtained by stomach flushing, following Xavier et al. [57] adapted to penguins (stomach samples were successfully obtained with a maximum of 3 flushes), following the Convention for the Conservation of Antarctic Marine Living Resources (CCAMLR) Ecosystem Monitoring Program (CEMP) Standard Methods. If the first flush produced a green or yellow coloured sample, it was assumed that the penguins were without food and they were released. All penguins handled were then marked to ensure they were sampled only once, and released. The procedures lasted, on average, 15 minutes.

The analyses of the food samples were carried out at the Bird Island research station laboratory within 24 hours of collection. Each food sample was analysed following Xavier et al. [58]. The samples were weighed and the overall mass was recorded. To remove the liquid, each food sample was washed carefully through two sieves (1.00 and 3.35 mm). Only fresh (recently consumed) material was found (i.e. no old cephalopod beaks nor other indigestible material (e.g. stones or plastics) was found). All components were then sorted into categories (crustaceans, fish, cephalopods and others; the latter comprising other fresh prey). Crustaceans were identified when possible using the BAS and Royal Belgium Institute of Natural Sciences reference collections, and the key reference bibliography of Kane [59] and Boltovskoy [60]. The total length of crustaceans was measured when possible (from tip of the eyes to the end of uropods/telson). The fish otoliths were identified following Hecht [61], Williams and McEldowney [62], Smale et al. [63], Reid [64] and fish size relationships used were given by Adams and Klages [65], Hecht [61], Williams and McEldowney [62], Smale et al. [63], Reid [64], Olsson and North [66], Berrow and Croxall [67] and Croxall et al. [68, 69]. We putatively identified one group of very small otoliths as? *Gymnoscopelus braueri* (preliminary identification suggested by Marcella Libertelli) but subsequent genetic analyses using flesh did not confirm this identification. The number of fish was estimated from the number of intact crania containing both otoliths, and loose otoliths, in each sample. These loose otoliths were compared with each other (right otolith compared with left otolith by size and level of erosion) and paired if possible (e.g. if four loose otoliths were found with similar sizes and similar sizes of erosion, and two were left otoliths and the other two were right otoliths, it was assumed that there had been two fish). The cephalopod beaks were counted (both upper and lower), identified and measured. Only the lower cephalopod beaks were measured, using the lower rostral length (LRL) following Xavier and Cherel [70] and the BAS beak reference collection. Allometric equations, of mantle length (ML) and estimated mass (W) for cephalopods were taken from Xavier and Cherel [70]. The components and species were analysed by frequency of occurrence, number and by mass following Xavier et al. [58].

### Stable isotopic analyses

Tissue δ^13^C and δ^15^N values provide useful information about seabird diet at different temporal scales [71]. The carbon stable isotope value (δ^13^C) mainly reflects consumers’ foraging habitat, while the nitrogen stable isotope value (δ^15^N) is mainly used to define consumers’ diet and trophic position. The isotopic niche of each adult was determined by using three complementary tissues (plasma, RBC and feathers) that record trophic information at different time scales [72]. Plasma and RBC retain information on diet (carbon source and trophic level) from a few hours/days prior to sample collection to the previous 3–4 weeks, respectively [73, 74]. Hence, the isotopic signature of plasma is representative of the food and feeding ecology of the penguins during their last few daily foraging trips. Breast feathers represent the diet during the previous pre-moulting stage, since feather keratin is metabolically inert after synthesis, which in Gentoo penguins from South Georgia occurs generally between March and April [75]. Therefore, based on stable isotopic analyses of different tissues from the same penguin we are able to test for short-term (i.e. days/weeks–RBC versus plasma) and medium-term (i.e. weeks to months–RBC versus feathers, when these were grown) consistency in individual foraging niche [56].

Lipids are depleted in ^13^C relative to whole tissues and were removed from plasma using successive rinses in a 2:1 chloroform: methanol solution [56, 76]. The low lipid content of whole blood (or RBC) does not typically require lipid extraction [77]. Prior to stable isotopic analyses, feathers were cleaned of surface contaminants using successive rinses in a 2:1 chloroform: ether solution, air-dried and then ground to a fine powder in a freezer mill operating at liquid nitrogen temperature.

Nitrogen and carbon isotope ratios were determined by a continuous-flow isotope ratio mass spectrometer (Delta V Advantage, Thermo Scientific) coupled to an elemental analyser (Flash EA1112, Thermo Scientific) in the LIENSs, Université de La Rochelle, France. Approximately 0.3 mg of each sample was combusted in a tin cup for the simultaneous determination of nitrogen and carbon isotope ratios. Results are presented in the usual δ notation based on the Vienna PeeDee Belemnite (V-PDB) for carbon and atmospheric N_2_ (AIR) for nitrogen. Replicate measurements of internal laboratory standards (acetanilide) indicate measurement errors <0.15 ‰ for both δ^13^C and δ^15^N.

To analyse stable isotope data in the context of isotopic niche between sexes, we adopted the recent metrics based in a Bayesian framework (Stable Isotope Bayesian Ellipses in R: SIBER [78]), which allows for robust statistical comparisons. The Bayesian approximation of the standard ellipse area (SEAb) is a metric used to test whether Group 1 (males) standard ellipse area (SEA) is smaller than Group 2 (females) and is calculated based on 1000 replications. The SEA corrected for small sample sizes (SEAc, an ellipse that contains 40% of the data regardless of sample size) was adopted to compare niche width between sexes (see Jackson *et al*. (2011) for more details). The SEAb and the layman metric of convex hull area (TA) [79] were also calculated as a measure of isotopic niche area. SEAb was used to test whether Group 1 is smaller than Group 2 (i.e. *p*, the proportion of ellipses in males that were lower than females), following Jackson *et al*. (2011). We used the computational code to calculate the metrics from SIBER implemented in the package SIAR [80] under R 3.2.1.

### Molecular sexing

Blood samples from Gentoo penguins were collected, as mentioned above, from 55 individuals to identify their sex. DNA from blood was isolated using an adaptation of the Chelex extraction method [81]. All samples were centrifuged for 3 min and a small portion of blood was removed for extraction with a spatula. 50 μl of distilled H_2_O and 20 μl of InstaGene^™^ Matrix (BioRad) were added to each sample. The samples were then incubated at 50°C for 30 min, followed by 8 min at 100°C. One negative control (a tube with all the reagents but without a blood sample) was included for each set of 24 extractions to monitor for possible contamination with exogenous DNA. Primers P2/P8 [82] were used for PCR amplification. These primers have been commonly used for penguins [83, 84] and provided sufficient separation of bands (~20 base pairs) to be differentiated on an agarose gel. All PCRs included two positive controls to test for the success of the amplification and two negative controls, prepared with distilled water, to test for possible contamination. Each male result was repeated at least three times and each female result was repeated at least twice. Amplifications were performed using a Multiplex kit, carried out in 10 μl reactions containing 1x of QIAGEN^®^ Multiplex PCR Master Mix, 0.2 μM of each primer and 0.8 μl of DNA template (~1 ng/μl). The thermal conditions were 95°C for 15 min, 35 cycles of 95°C for 1 min, 47°C (annealing temperature) for 1 min 30 s, 72°C for 1 min 30 s, and a final extension at 72°C for 10 min. All reactions were carried out using an Applied Biosystems Veriti^®^ Thermal Cycler PCR machine. Samples were run for about 2h on 3% weight/volume agarose gels stained with ethidium bromide.

### Dead counts

In September 2009 (1^st^ and 8^th^ Sept.), two surveys for dead Gentoo penguins (n = 111) were carried out at Bird Island (at Landing Beach, Iceberg Point, Freshwater Beach, Stinker Point and Everman Cove), due to the appearance of numerous dead Gentoo penguins on the beaches of Bird Island, South Georgia. After identifying a body of a Gentoo penguin, the bill length and bill depth were measured, as above. Sex was estimated using equations from Williams [85]. It was also reported if it was recently dead or an old carcass. To avoid duplicating the identification of dead animals in the second survey, stock marker was used to paint the bill of the penguins.

Data were statistically analysed using Minitab statistical software (Sowers Printing Company, PA, USA) and R [86]. The values are as mean ± standard deviation, unless stated (significance threshold: 0.05).

## Results

The feeding ecology of 55 Gentoo penguins from Bird Island (South Georgia) was studied during the austral winter of 2009 (Table 1; S1 Dataset). Of these, 12 had empty stomachs (or only minor residues of food), which were removed from further analyses. Based on individuals whose stomach contents were analysed, our study showed that male Gentoo penguins (N = 17; body mass = 6666 ± 653 g) were significantly heavier than females (N = 26; body mass = 5334 ± 520 g; Mann-Witney U test, U = 409, P < 0.01). The mean solid proportion of Gentoo penguin food samples ranged from 4 to 414 g (141 ± 101 g), with females having significantly more solids than males (Mann-Witney U test, U = 114, P < 0.01).

**Table 1:** Parameters collected from Gentoo penguins during winter 2009 at Bird Island, South Georgia (F = Female, M = Male).

		Bill length (mm)		Bill depth (mm)		Penguin size (cm)		Penguin mass (g)		Sample solids (g)	
Sex	n	Mean ± SD	Range	Mean ± SD	Range	Mean ± SD	Range	Mean ± SD	Range	Mean ± SD	Range
F	30	49.1±0.4	44.4–54.4	15.5±0.1	13.5–17.0	42.0±0.2	39.0–44.0	5277±98	4350–6380	172.2±19.6	24.5–413.7
M	25	53.2±0.5	49.1–60.0	17.2±0.2	15.3–18.6	44.8±0.3	41.7–48.0	6524±127	5050–7500	92.7±20.5	4.4–297.8
F+M	55	50.9±0.4	44.4–60.0	16.3±0.2	13.5–18.6	43.3±0.3	39.0–48.0	5844±115	4350–7500	140.8±15.4	4.4–413.7

### Diet composition

**Fig 3:**
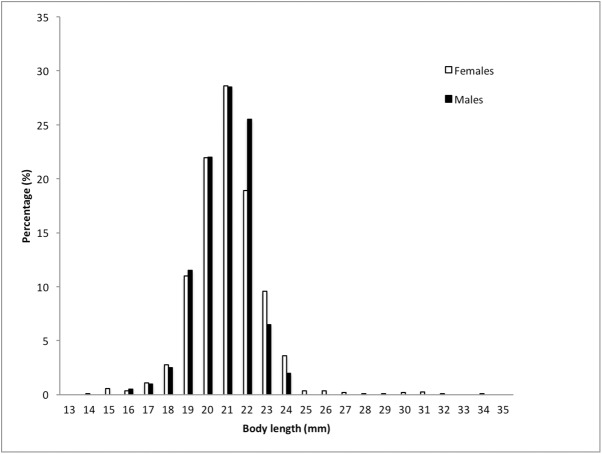
Length frequency distribution of *Themisto gaudichaudii* from the diet of Gentoo penguins (females and males) obtained at Bird Island, South Georgia in winter 2009.

Overall, when samples for both sexes were combined, Gentoo penguins fed primarily on crustaceans (68% by mass) followed by fish (25%; Table 2). Cephalopods and other prey/debris represented <1% and 7% by mass, respectively. By prey species, *Themisto gaudichaudii* was the most important crustacean by frequency of occurrence (74%), by number (77%) and by mass (63%; Table 2). The proportion of crustaceans and fish consumed differed significantly between sexes (i.e. males consumed more fish (Mann-Witney U test, U = 348, P < 0.01) and females more crustaceans (Mann-Witney U test, U = 26, P < 0.01), see below), whereas the proportion of cephalopods (Mann-Witney U test, U = 242, P = 0.6) and other prey (Mann-Witney U test, U = 212, P = 0.8) did not differ. Prey sizes of Gentoo penguins ranged from 5.6 mm total length (?*Gymnoscopelus braueri*) to 447 mm (*Parachaenychthis georgianus*; Table 3), with females eating bigger crustaceans (Mann-Witney U test, U = 157963, P < 0.01) and fish (Mann-Witney U test, U = 18034, P < 0.01) than male penguins (Table 3; see below). However, by comparing sizes (total length) of prey between penguin sexes (with ≥ 10 individual prey in both sexes), *T*. *gaudichaudii* had similar sizes for both sexes (Mann-Witney U test, U = 118166, P = 0.70; Fig 3), as well as *Champsocephalus gunnari* (Mann-Witney U test, U = 307, P = 0.98), *Lepidonotothen larseni* Mann-Witney U test, U = 364, P = 0.25), and? *Gymnoscopelus braueri* (Mann-Witney U test, U = 407, P = 0.44). Only female Gentoo penguins fed on significantly bigger *Muraenolepis microps* than males (Mann-Witney U test, U = 423, P < 0.01).

**Table 2:** Number of samples, frequency of occurrence (F), number of crustaceans/fish/cephalopods (N), and mass (M; with SD for main components). Percentages of the species were calculated within each main diet component, collected from female (26 samples) and male (17 samples) Gentoo penguins.

		Overall			Females			Males	
	F (%)	N (%)	M (%)	F (%)	N (%)	M (%)	F (%)	N (%)	M (%)
**Crustaceans**	**86.0**	**98.4**	**68.4±16.0**	**80.8**	**99.0**	**88.7±21.5**	**58.8**	**89.1**	**37.5±40.8**
***Amphipoda***									
Ampeliscidae									
*Byblis securiger*	16.3	0.1	0.3	26.9	0.1	0.3	<0.1	<0.1	<0.1
Eurytheneidae									
*Eurythenes gryllus*	2.3	<0.1	<0.1	3.8	<0.1	<0.1	0.0	0.0	0.0
Hyperiidae									
Hyperiidae sp.	2.3	<0.1	<0.1	3.8	<0.1	<0.1	0.0	0.0	0.0
*Themisto gaudichaudii*	74.4	76.9	62.8	88.5	76.7	62.7	52.9	81.5	63.5
Oedicerotidae									
*Oediceroides* cf. *lahillei*	2.3	<0.1	<0.1	3.8	<0.1	<0.1	0.0	0.0	0.0
Pontogeneiidae									
*Djerboa furcipes*	2.3	<0.1	<0.1	3.8	<0.1	<0.1	0.0	0.0	0.0
*Eusiroides* sp.	2.3	<0.1	<0.1	3.8	<0.1	<0.1	0.0	0.0	0.0
*Gondogeneia antarctica*	2.3	<0.1	<0.1	3.8	<0.1	<0.1	0.0	0.0	0.0
*Gondogeneia georgiana*	18.6	10.9	4.6	26.9	11.4	4.8	5.9	0.4	0.2
*Paramoera walkeri*	2.3	<0.1	<0.1	3.8	<0.1	<0.1	0.0	0.0	0.0
Pontogeneiidae sp.	2.3	<0.1	<0.1	3.8	<0.1	<0.1	0.0	0.0	0.0
Euchaetidae									
*Euchaeta* sp.	2.3	<0.1	<0.1	3.8	<0.1	<0.1	0.0	0.0	0.0
Lysianassidae									
*Orchomenopsis acanthura*	4.7	<0.1	<0.1	7.7	<0.1	<0.1	0.0	0.0	0.0
Vibiliidae									
*Vibilia antarctica*	9.3	1.3	0.4	15.4	1.4	0.4	0.0	0.0	0.0
***Decapoda***									
Crangonidae									
*Notocrangon antarcticus*	32.6	0.2	0.5	30.8	0.1	0.2	35.3	1.6	5.7
Decapoda undet.	2.3	<0.1	<0.1	3.8	<0.1	<0.1	0.0	0.0	0.0
**Euphausiacea**									
Euphausiidae									
*Euphausia frigida*	14.0	<0.1	0.1	19.2	<0.1	0.1	5.9	0.1	<0.1
*Euphausia superba*	65.1	6.2	19.8	76.9	5.8	19.2	47.1	14.2	29.1
*Euphausia triacantha*	2.3	<0.1	<0.1	3.8	<0.1	<0.1	5.9	<0.1	<0.1
Euphausiidae sp.	4.7	<0.1	<0.1	3.8	<0.1	<0.1	5.9	0.1	<0.1
*Thysanoessa* sp.	39.5	2.6	3.4	57.7	2.6	3.5	11.8	2.0	1.2
**Isopoda**									
Serolidae									
*Serolis bouvieri*	2.3	<0.1	<0.1	3.8	<0.1	<0.1	0.0	0.0	0.0
**Lophogastrida**									
Gnathophausiidae									
*Gnathophausia* sp.	2.3	<0.1	<0.1	3.8	<0.1	<0.1	0.0	0.0	0.0
**Mysida**									
Mysidae									
*Antarctomysis maxima*	30.2	1.7	8.1	42.3	1.7	8.5	11.8	0.2	0.1
**Fish**	**88.4**	**1.5**	**24.9±38.3**	**96.2**	**1.0**	**3.7±8.0**	**76.5**	**10.6**	**57.4±43.4**
Bathydraconidae									
*Parachaenichthys georgianus*	4.7	10.1	46.3	0.0	0.0	0.0	11.8	26.6	55.3
Channichthyidae									
*Champsocephalus gunnari*	37.2	15.8	24.5	46.2	19.2	66.8	23.5	10.1	16.3
Channichthyidae	2.3	0.3	<0.1	0.0	0.0	0.0	5.9	0.7	<0.1
*Pseudochaenichthys georgianus*	4.7	0.5	1.4	3.8	0.4	4.1	5.9	0.7	0.9
Myctophidae									
?*Gymnoscopelus braueri*	48.8	38.6	0.6	65.4	59.4	3.6	23.5	4.3	0.1
*Krefftichthys anderssoni*	4.7	0.5	<0.1	3.8	0.4	<0.1	5.9	0.7	<0.1
Muraenolepididae									
*Muraenolepis microps*	44.2	12.8	1.4	50.0	13.1	1.3	35.3	12.2	1.4
Nototheniidae									
*Gobionotothen gibberifrons*	4.7	0.5	8.7	0.0	0.0	0.0	11.8	1.4	10.3
*Lepidonotothen larseni*	30.2	18.2	12.4	23.1	6.6	24.2	47.1	37.4	10.1
*Trematomus hansoni*	2.3	0.5	4.7	0.0	0.0	0.0	11.8	1.4	5.7
Unknown fish (Osteichthyes)	14.0	2.2	<0.1	7.7	0.9	<0.1	23.5	4.3	<0.1
**Cephalopods**	**11.6**	**<0.1**	**0.1±0.0**	**11.5**	**<0.1**	**<0.1±0.2**	**11.8**	**0.2**	**0.1±0.4**
Brachioteuthidae									
*Slosarczykovia circumantarctica*	7.0	66.7	75.9	3.8	66.7	45.9	11.8	66.7	100.0
Onychoteuthidae									
*Kondakovia longimana*	4.7	33.3	24.1	3.8	33.3	54.1	5.9	33.3	<0.1
**Others**	**11.6**	**<0.1**	**6.6±16.0**	**19.2**	**0.1**	**7.6±19.6**		**0.0**	**5.0±8.0**
Sagittidae									
*Sagitta* sp.	11.6		1.2	19.2		2.5	0.0		0.0
*Debris/stones/unidentified material*	44.2		98.8	42.3		97.5	47.1		100.0

**Table 3:** Measurements from crustaceans, fish and cephalopods obtained from the diet of Gentoo penguins.

Crustaceans	Sex	n	CL Mean (range)	TL Mean (range)	Mass Mean (range)
*Antarctomysis maxima*	F	101	8.4 (4.8–12.0)	33.1 (19.0–47.0)	n/a
	M	0			
	F+M	101	8.4 (4.8–12.0)	33.1 (19.0–47.0)	n/a
*Byblis securiger*	F	13	n/a	37.1 (34.0–39.0)	n/a
	M	0			
	F+M	13	n/a	37.1 (34.0–39.0)	n/a
*Euphausia frigida*	F	3	n/a	18.3 (12.0–23.0)	n/a
	M	1	n/a	23.0	n/a
	F+M	4	n/a	19.5 (12.0–23.0)	n/a
*Euphausia superba*	F	254	n/a	44.5 (27.0–58.0)	n/a
	M	19	n/a	45.7 (40.0–55.0)	n/a
	F+M	273	n/a	44.6 (27.0–58.0)	n/a
*Euphausia triacantha*	F	1	n/a	26.0	n/a
	M	0			
	F+M	1	n/a	26.0	n/a
*Eurythenes gryllus*	F	2	n/a	25.0 (24.0–26.0)	n/a
	M	0			
	F+M	2	n/a	25.0 (24.0–26.0)	n/a
*Gondogeneia georgiana*	F	13	n/a	18.3 (15.0–21.0)	n/a
	M	0			
	F+M	13	n/a	18.3 (15.0–21.0)	n/a
*Notocrangon antarcticus*	F	8	n/a	37.3 (32.0–42.0)	n/a
	M	1	n/a	20.0	n/a
	F+M	9	n/a	35.3 (20.0–42.0)	n/a
*Themisto gaudichaudii*	F	1202	n/a	22.0 (15.0–35.0)	n/a
	M	200	n/a	21.9 (17.0–25.0)	n/a
	F+M	1402	n/a	22.0 (15.0–35.0)	n/a
*Thysanoessa* sp.	F	185	n/a	28.0 (15.0–35.0)	n/a
	M	0			
	F+M	185	n/a	28.0 (15.0–35.0)	n/a
*Vibilia antarctica*	F	1	n/a	12.0	n/a
	M	0			
* *	F+M	1	n/a	12.0	n/a
Fish			OL Mean (range)		
*Champsocephalus gunnari*	F	77	1.1 (0.7–1.9)	142.3 (89.4–230.8)	17.1 (3.1–74.1)
	M	25	1.4 (0.9–3.4)	168.5 (113.5–401.2)	67.7 (6.9–472.3)
	F+M	102	1.2 (0.7–3.4)	148.3 (89.4–401.2)	29.5 (3.1–472.3)
*Gobiotothen gibberifrons*	F	0			
	M	2	7.4 (6.8–8.0)	318.5 (285.5–351.5)	300.3 (199.4–401.1)
	F+M	2	7.4 (6.8–8.0)	318.5 (285.5–351.5)	300.3 (199.4–401.1)
*Krefftichthys anderssoni*	F	2	0.9	34.7	0.4
	M	1	0.7	23.1	0.2
	F+M	3	0.8 (0.7–0.9)	28.9 (23.1–34.7)	0.3 (0.2–0.4)
*Lepidonotothen larseni*	F	26	3.2 (0.9–5.1)	82.7 (38.7–181.2)	18.7 (0.5–54.7)
	M	82	2.6 (0.9–4.7)	83.7 (38.7–168.5)	11.7 (0.5–43.7)
	F+M	108	2.7 (0.9–5.1)	89.1 (38.7–181.2)	13.5 (0.5–54.7)
*Muraenolepis microps*	F	52	1.1 (0.7–2.5)	34.3 (15.9–121.8)	0.5 (<0.1–11.1)
	M	33	1.7 (1.0–3.2)	69.8 (28.1–180.8)	4.8 (0.1–42.5)
	F+M	85	1.3 (0.7–3.2)	47.1 (15.9–180.8)	2.0 (<0.1–42.5)
*Parachaenychthis georgianus*	F	0			
	M	64	2.4 (1.7–5.3)	198.9 (143.3–446.7)	86.8 (35.9–469.5)
	F+M	64	2.4 (1.7–5.3)	198.9 (143.3–446.7)	86.8 (35.9–469.5)
*Pseudochaenichthys georgianus*	F	2	1.9	160.1	46.2
	M	1	2.0	168.6	51.8
	F+M	3	1.9 (1.9–2.0)	164.3 (160.1–168.6)	49.0 (46.2–51.8)
*Trematomus hansoni*	F	0			
	M	4	4.9 (4.8–5.0)	249.5 (244.4–254.7)	164.5 (152.6–176.3)
	F+M	4	4.9 (4.8–5.0)	249.5 (244.4–254.7)	164.5 (152.6–176.3)
*Gymnoscopelus braueri*	F	260	0.8 (0.4–1.5)	27.1 (5.6–69.5)	0.3 (0.1–2.4)
	M	10	0.9 (0.4–1.3)	34.7 (5.6–57.9)	0.7 (0.1–1.3)
	F+M	270	0.8 (0.4–1.5)	27.3 (5.6–69.5)	0.3 (0.1–2.4)
Cephalopods			LRL Mean (range)	ML Mean (range)	
*Kondakovia longimana*	F	1	1.1	18.7	2.5
	M	yes (upper beak)			
	F+M	2	1.1	18.7	2.5
*Slosarczykovia circumantarctica*	F	2	0.7	30.4	1.0
	M	2	1.4 (1.0–1.8)	44.6 (36.5–52.6)	2.9 (1.7–4.0)
	F+M	4	1.1 (0.7–1.8)	37.5 (30.4–52.6)	1.9 (1.0–4.0)

(LRL = Lower rostral length (mm); OL = Otolith length (mm); CL = Carapace length (mm); TL = Total length (mm); ML = Mantle length (mm); F = Female; M = Male) (n/a = not applicable)

The mean solid proportion of male Gentoo penguin food samples ranged from 4 to 298 g (93 **±** 84 g). Males fed mainly on fish (54% by mass) followed by crustaceans (38%), other species (5%) and cephalopods (< 1%). *T*. *gaudichaudii* was the most important crustacean prey (64% by mass; 82% by number; 53% by frequency of occurrence) (Table 1). *Euphausia superba* represented only 29% by mass and 14% by number, although it had 47% by frequency of occurrence (Table 1). Within the fish component, *P*. *georgianus* was the most important fish prey by mass (55% by mass; 27% by number; 12% by frequency of occurrence) and *L*. *larseni* by number and frequency of occurrence (10% by mass; 37% by number; 47% by frequency of occurrence; Table 1).

The mean solid proportion of female Gentoo penguins food samples ranged from 25 to 414 g (172 **±** 100 g), significantly heavier than males (see above). Females fed mainly on crustaceans (89% by mass) followed by fish (4%), others (8%) and cephalopods (< 1%). *T*. *gaudichaudii* was the most important crustacean prey (63% by mass; 77% by number; 89% by frequency of occurrence), followed by *E*. *superba* (19% by mass; 6% by number; 77% by frequency of occurrence). Of the fish, *C*. *gunnari* (67% by mass; 19% by number; 46% by frequency of occurrence) was the most important fish prey by mass (Table 1). *P*. *georgianus* (the most important fish species in male Gentoo penguins) was absent in female Gentoo penguins (Table 1).

### Stable isotopic and niche analyses

Stable isotopic and niche analyses, both δ^13^C and δ ^15^N, were carried out on RBC, plasma and breast feathers of male and female Gentoo penguins (all individuals; n = 55) (Table 4). Overall, the values in terms of δ^13^C in Gentoo penguins ranged between -21.0 and -17.8 ‰ (blood cells: between -21.0 and -18.7 ‰ δ ^13^C; plasma: between -21.3 and -18.0 ‰ δ ^13^C; feathers: -21.0 and -17.8 ‰ δ ^13^C) whereas in terms of δ^15^N, the values ranged between 8.6 and 15.1 ‰ (blood cells: between 9.8 and 14.1 ‰ δ ^15^N; plasma values: between 10.5 and 15.1 ‰ δ ^15^N; feathers: varied between 8.6 and 13.6 ‰ δ ^15^N) (Table 4).

**Table 4:** Stable isotopic values of plasma, red blood cells and feathers from female (F) and male (M) Gentoo penguins.

Temporal integration	Plasma (few days)			Red blood cells (few weeks)			Feathers (March- April, when producing them)		
Sex	F	M	F+M	F	M	F+M	F	M	F+M
n	30	25	55	30	25	55	30	25	55
δ^13^C	-20.1±0.5	-19.6±0.8	-19.9± 0.7	-20.1 ± 0.4	-19.8±0.5	-20.0±0.5	-19.5±0.5	-20.0±0.8	19.7±0.7
Range	(-21.3; -19.5)	(-20.8; -18.0)	(-21.3; -18.0)	(-21.0; -19.4)	(-20.7; -17.5)	(-21.0; -18.7)	(-20.6; -18.5)	(-21.0; -17.8)	(-21.0; -17.8)
δ^15^N	11.7±0.5	13.3±1.2	12.4±1.2	10.8±0.5	12.1±1.0	11.4±1.0	11.5±1.0	11.8±1.1	11.7±1.0
Range	(10.5; 13.2)	(11.1; 15.1)	(10.5; 15.1)	(9.8; 11.8)	(10.4; 14.1)	(9.8; 14.1)	(8.6; 13.2)	(9.6; 13.6)	(8.6; 13.6)
C:N mass ratio	3.5±0.1	3.4±0.1	3.4±0.1	3.3±0.1	3.3±0.1	3.3±0.1	3.2±0.0	3.2±0.0	3.2±0.0

### Sexual differences

In terms of foraging habitat (δ^13^C), there were significant differences between sexes in plasma values, with males having higher plasma δ ^13^C values (Mann-Witney U test, U = 471, P <0.05), but not in RBC values (Mann-Witney U test, U = 380, P = 0.57) (Table 4). Feather δ^13^C values showed that females had significantly higher δ^13^C values than males (Mann-Witney U test, U = 172, P <0.01).

In terms of trophic level (δ^15^N), there were significant differences between sexes in plasma values (Mann-Witney U test, U = 618, P <0.01) and in RBC values (Mann-Witney U test, U = 626, P < 0.01), with males having higher values (Table 4). Feather δ^15^N values showed no sex-related differences (Mann-Witney U test, U = 384, P = 0.52; Table 4).

There were also positive significant relationships between isotopic values of δ^13^C and δ^15^N, both in plasma and RBC, with relationships significantly higher in males (δ^13^C versus δ^15^N in RBC: Pearson correlation 0.92, P< 0.01; plasma: Pearson correlation 0.92, P< 0.01) than for females (RBC: Pearson correlation 0.54, P< 0.01; plasma: Pearson correlation 0.43, P = 0.02). However, no significant relationships between isotopic values of δ^13^C and δ^15^N in feathers were found in males (Pearson correlation 0.08, P = 0.18) or females (Pearson correlation 0.12, P = 0.06).

**Fig 4:**
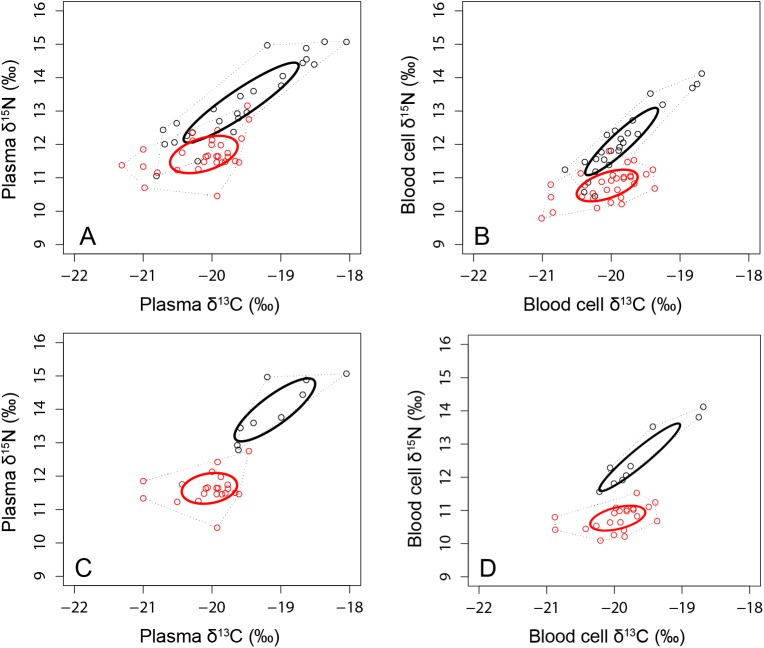
Isotopic niche area based on stable isotope values (δ^13^C and δ^15^N) in plasma and Red blood cells of male (*black*) and female (*red*) Gentoo penguins breeding at Bird Island in winter 2009 (A and B), and individuals showing prey preference (C and D, i.e. males with > 80% fish and females with > 80% krill in their stomachs). The area of the standard ellipses (SEAc, 40% credible interval) was represented by the solid bold lines (ellipses), and the Layman metric of convex hull area (TA) by black dotted lines (see Jackson *et al*. (2011) for more details on these metrics of isotopic niche width).

The isotopic niche width (SEAb) was estimated and found to be significantly higher in males than in females using plasma (p = 0.01) and RBC (p = 0.04), and practically no overlap was detected in the isotopic data (i.e. the area of the standard ellipses; SEAc) of males and females, with males having higher levels of δ^15^N (Table 5; Fig 4). On the other hand, although SEAb was also higher in males than in females using feathers (p = 0.05), a relatively high overlap was detected in the isotopic niche between sexes, with overall larger areas than in blood tissues (Table 5).

**Table 5:** Metrics of isotopic niche width in plasma and red blood cells’ tissues of Gentoo penguins obtained at Bird Island, South Georgia in winter 2009. The area of the standard ellipse (SEAc), the layman metric of convex hull area (TA) and the overlap between males and females for each metric were calculated from SIBER (see Jackson et al. 2011 for more details on these metrics).

	SEAc	Overlap (SEAc)	TA	Overlap (TA)
**Plasma**				
Overall (n = 55)	1.58	-	6.40	-
Males (n = 25)	1.23	< 0.01	3.71	1.12
Females (n = 30)	0.78		2.79	
**Red blood cells**				
Overall (n = 55)	1.08	-	4.21	-
Males (n = 25)	0.65	< 0.01	2.05	0.57
Females (n = 30)	0.56		1.93	
**Feathers**				
Overall (n = 55)	2.14	-	9.15	-
Males (n = 25)	2.58	0.95	7.24	4.79
Females (n = 30)	1.51		6.22	

### Individual specialization

**Fig 5:**
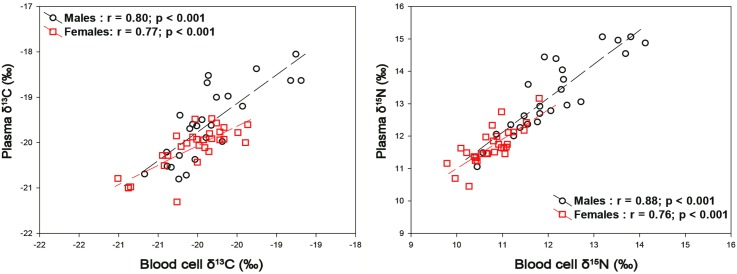
Individual relationships of stable isotope values (δ^13^C and δ^15^N) in plasma and Red blood cells of male (*black*) and female (*red*) Gentoo penguins breeding at Bird Island in winter 2009.

**Fig 6:**
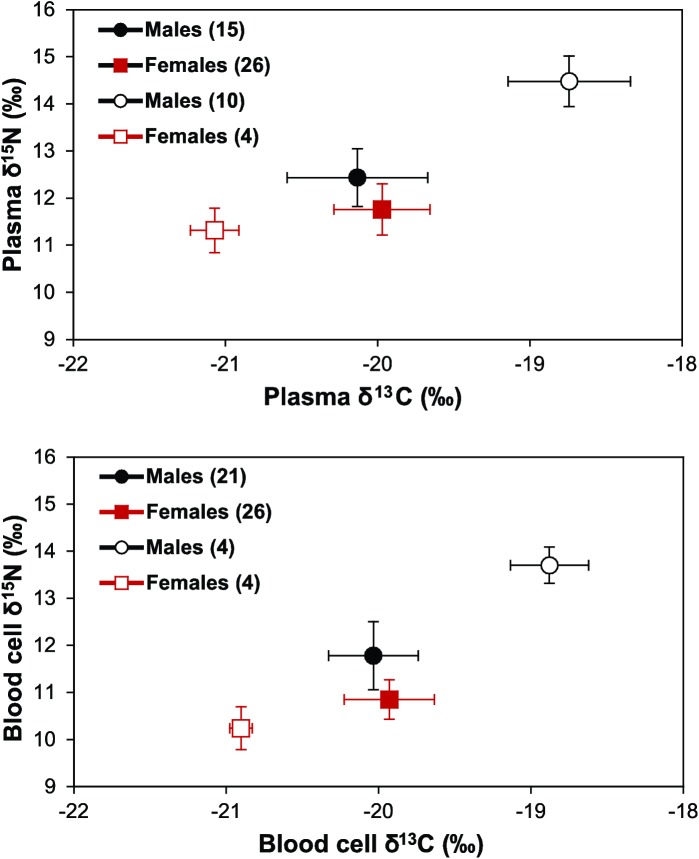
Stable isotope values of carbon (δ^13^C) and nitrogen (δ^15^N) (means ± SD) in plasma and red blood cells of male and female Gentoo penguins breeding at Bird Island in winter 2009 (the number of individuals used are in brackets, which were grouped according to their different values of carbon and nitrogen).

Both males and females exhibited individual specialization (Figs 4,5 and 6). A strong positive relationship relating δ^13^C in RBC versus plasma was found for both males (Pearson correlation 0.80, P< 0.01) and females (Pearson correlation 0.77, P< 0.01) (Fig 5). Similarly, when relating δ^15^N in RBC versus plasma, a strong positive relationship was found for both males (Pearson correlation 0.88, P< 0.01) and females (Pearson correlation 0.76, P< 0.01) (Fig 5). Furthermore, a group of female individuals (n = 4) segregated with lower δ^13^C and δ^15^N from most females whereas a group of male individuals (of variable number) clearly segregated with higher δ^13^C and δ^15^N from most males (Figs 4 and 6). Also, when assessing individuals with highly divergent diets, male individuals that fed on > 80% by mass on fish and compared with females that fed on > 80% by mass on Antarctic krill, the differences in δ^15^N is even more obvious (Fig 4).

Finally, when relating δ^13^C in feathers versus RBC, no relationships were found for either males (Pearson correlation 0.22, P = 0.29) or females (Pearson correlation 0.24, P = 0.24). Similar results were found when assessing δ^15^N in feathers versus RBC, with no relationships found for either males (Pearson correlation 0.26, P = 0.21) or females (Pearson correlation 0.01, P = 0.95).

### Dead counts

A total of 111 individual dead Gentoo penguins (of which 34 individuals were classified as recently dead) were found along Bird Island beaches in the two surveys. The bill length measurements were from 42.3 to 57.2 mm (50.7 ± 0.4 mm) and the bill depth between 13.3 to 18.6 mm (16.0 ± 0.1 mm), with females and males representing 69.8% and 30.2% of the penguins, respectively (n = 86 of penguins with measurable bills). There were no differences between the dead penguins and studied penguins in bill length measurements (Mann-Witney U test, U = 1813, P = 0.86) nor bill depth measurements (Mann-Witney U test, U = 2011, P = 0.42). No bodies were identified as being attacked by leopard seals *Hydrurga leptonyx* or other predators.

## Discussion

Our study showed that the feeding ecology of Gentoo penguins during their winter non-breeding season, under abnormal environmental conditions, presented significant differences in sexual and individual segregation between males and females, with males feeding more on fish and females more on crustaceans. Further evidence came from differences noted both in terms of foraging habitat (δ^13^C values; using plasma and feathers) and trophic level (δ^15^N; using plasma and red blood cells). Individual specialization was also detected with individual males and individual females segregating from the majority of the individuals. Such levels of sexual and individual segregation, in terms of diet, foraging habitat and trophic level under abnormal environmental conditions, related to a bias of more female dead individuals, may have a negative impact in the population of local Gentoo penguin populations.

### Characterization of the diet of Gentoo penguins during the austral winter at South Georgia

Gentoo penguins at South Georgia during the austral winter feed mainly on crustaceans and fish (our study; Kato et al. 1991; Williams 1991; Williams *et al*. 1992) (Table 6). Within the crustacean component, our study showed that Gentoo penguins at South Georgia fed more on *Themisto gaudichaudii* (63% by mass,) rather than Antarctic krill *Euphausia superba* (20% by mass), contrasting with previous studies [26–28]. These previous studies have shown that Antarctic krill dominated the diet in winter 1987 and 1988 (> 87% by mass). In 1989, fish (not identified to species level) dominated the first sampling period, in early July (73–76% by mass) and Antarctic krill dominated the second sampling period, in late July (> 89% by mass) [27]. Williams *et al*. [26] also conducted their study in late July, with Antarctic krill also dominating the diet (63.3–100.0% by mass).

**Table 6:** Diets of Gentoo penguins at South Georgia according to poor/good Antarctic krill *Euphausia superba* years. (n = number of samples).

		Diet (% by Mass)		Antarctic	
Year	Season	Females	Males	Krill abundance	References
1976/1977	Summer	70.1% Antarctic krill/32.6% fish (n = 43)		?	[90]
1985/1986	Summer	51.4% Antarctic krill/48.5.% fish (n> 100)		Low?	[20, 88, 89]
1987	Winter	87% crustaceans (n = 20)	83% fish (n = 13)	?	[28]
1988	Winter	> 87% Antarctic krill (n = 68)		?	[28]
1989	Winter	27–99% Crustaceans (n = 36)	24–89% Crustaceans (n = 33)	?	[27]
		0.1–73% Fish (n = 9)	11–76% Fish (n = 9)	?	
1993/1994	Summer	85.9% Fish (n = ?)		Low	[20]
1996	Winter	36.3–95.2% Antarctic krill (n = 48)		?	[87]
1996/1997	Summer	42.6–61.3% Antarctic krill (n = 46)		High	[45, 87]
2009	Winter	89% Crustaceans/4% Fish (n = 26)	54% Fish/38% Crustaceans (n = 17)	Low?	Present study

The variation in diets of Gentoo penguins at South Georgia have been linked to food availability locally [26–28], as Gentoo penguins are inshore-feeding species. Under the context of low Antarctic krill availability in autumn 2009 [44, 46], the diet shift of Gentoo penguins to *T*. *gaudichaudii* implies that high abundances of Antarctic krill did not occur during the following austral winter 2009 in inshore areas where Gentoo penguins forage. Moreover, the occurrence of Gentoo penguins found dead on the beaches (potentially a seabird wreck; see [47]) may support such a statement, although we need to be cautious as no counts of dead penguins from previous years are available, nor their cause of death. From other data available, it suggests that in years of good Antarctic krill availability, Gentoo penguins do feed mainly on Antarctic krill (Table 6). In years of low Antarctic krill availability, the importance of fish increases and the crustacean component is replaced by *T*. *gaudichaudii* or other (not Antarctic krill) crustaceans [20, 45, 87–90] (Table 6).

### Sexual foraging segregation and individual specialization in Gentoo penguins

To our knowledge, this is the first study to assess sexual dietary differences in austral winter under known (abnormal) environmental conditions, when Gentoo penguins are not constrained by breeding duties. Female Gentoo penguins had significantly heavier meals (i.e. solids) than males (see Results) but as prey were inadequate (i.e. the availability of *T*. *gaudichaudii* was low), this fact was reflected in females struggling during austral winter 2009; with more dead female Gentoo penguins found than males on shores (see Results).

Although both sexes of Gentoo penguins are known to forage in inshore waters [25], males exhibited a different diet in comparison with females (Tables 1, 4 and 7): sexual differences in Gentoo penguins were found, with females taking more crustaceans (i.e. *T*. *gaudichaudii*) and males more fish. Also, Bearhop *et al*. [91] found that Gentoo penguin males foraged at a slightly higher trophic level than females (although not statistically significant), during the austral summer. Therefore, the “Intersexual Competition Hypothesis” for dimorphic species, does apply to Gentoo penguins. This is potentially due to the ability of male Gentoo penguins, with slightly larger bills compared to females (and also heavier mass; see Results) being able to catch fish prey when confronted with low availability of Antarctic krill around South Georgia during their non-breeding period [26–28], as suggested for other penguins elsewhere [92, 93]. Moreover, male Gentoo penguins are larger, heavier and are able to go deeper to forage than females [94, 95], and are able to feed on more bentho-pelagic prey (i.e. *Lepidonotothen larseni*, *Parachaenichthys georgianus* [96, 97]) than females, which tend to be limited to shallower pelagic waters. However, Croxall *et al*. [89] and Bost *et al*. [98] found no significant differences in the diving parameters of male and female Gentoo penguins. With such flexibility in their diet and in foraging habitat, male Gentoo penguins may be more resilient, especially in years when pelagic prey (i.e. Antarctic krill, *T*. *gaudichaudii*) are generally scarce (see below); this interpretation is also supported by our results in finding more females dead on the beaches than males (see results). Volkman et al. [99] also showed that males fed significantly more on fish than female Gentoo penguins, breeding at King George Island, despite the lack of differences in energy expenditure or foraging trip duration demonstrated for this species [75]. This supports the theory that sex differences in dietary preference changes of Gentoo penguins may largely reflect local changes in the availability of particular prey species within the inshore area exploited by this all-year-round predator, where intraspecific competition is intense (particularly during the winter when food availability is low). To avoid competition, Gentoo penguins switched their foraging behaviour by foraging at deeper depths to limit competition with Adélie penguins *Pygoscelis adeliae* at West Antarctic Peninsula [23].

**Table 7:** Studies that provide evidence on sexual segregation (by diet, prey size or foraging) and individual specialization in penguins.

		Sexual Segregation				
Penguin Species	Breeding cycle	Diet	Prey size	Foraging	Ind. specialization	References
Adélie penguins	Breeding season (Summer)	Yes	Yes	?		[99]
Adélie penguins	Breeding season (Summer)	Yes	?	Yes/No	Site fidelity (feeding area specialization– 63% of birds)	[100, 101, 110, 111]
Chinstrap penguins	Breeding season (Summer)	No	No	?		[99]
Chinstrap penguins	Breeding season (Summer)	Yes	?	?		[112]
Emperor penguins	Breeding season (Winter)	Yes	No?	Yes		[104]
Gentoo penguins	Non-breeding season (Winter)	Yes	No	?		Present study, [26–28]
Gentoo penguins	Breeding season (Summer)	Yes	?	No		[91]
Gentoo penguins	Breeding season (Summer)	Yes	No	?		[99]
Gentoo penguins	Breeding season (Summer)	Yes	?	?	Diet (related to mercury levels)	[40, 113]
Gentoo penguins	Breeding season (Summer)	Yes	?	?		[112]
Humboldt penguins	Breeding season (Winter)	?	?	Yes/No		[105]
King penguins	Breeding season (Summer)	?	?	?	Short-term consistency in the foraging niches	[114]
Little penguins	Breeding season (Summer)	?	?	Yes	Diving behaviour	[115–117]
Macaroni penguins	Breeding season (Summer)	No	?	Yes/No	Short-term specialization in the foraging niche during Winter	[38, 75, 91, 118]
Magellanic penguins	Breeding season (Summer)	Yes	?	?		[92]
Rockhopper penguins	Breeding season (Summer)	Yes	?	?		[93, 102, 103]
Rockhopper penguins	Breeding season (Summer)	?	?	No		[119]
Royal penguins	Breeding season (Summer)	?	?	No		[119]
Yellow-eyed penguins	Breeding season (Summer)	?	?	No		[120]

In comparison with other penguin species, comparable diet and foraging data is mostly available for the summer breeding season (Table 7). Adélie penguins [99–101], southern rockhopper penguins *Eudyptes chrysocome* [93, 102, 103], Magellanic penguins *Spheniscus magellanicus* [92], Emperor penguins *Aptenodytes forsteri* [104] are also known to exhibit sex differences in trip duration, foraging areas and diets. With these data, it is not clear why these patterns vary across species (Tables 6 and 7), but differences during the breeding period in some species (e.g. Emperor and Humboldt penguins *Spheniscus humboldti*) may be related to body condition, and the role of each sex (e.g. females must produce the egg, whereas Emperor penguin males are solely responsible for incubating the egg) [104, 105].

In terms of habitat use (based on δ^13^C), there were variable results in short-term carbon signatures between males and females of Gentoo penguins (based on plasma and red blood cells), emphasising a changeable habitat use through the austral winter. Such a result is possibly a reflection of male and female Gentoo penguins trying to avoid (when possible) competition for the same prey (due to the lack of prey availability locally), and thus broadening their habitat use.

In terms of the trophic level (based on δ^15^N), males fed significantly at higher trophic levels than females (based on plasma and red blood cells; Figs 4 and 5), confirmed by male Gentoo penguins feeding on bentho-pelagic/benthic high trophic level prey (i.e. fish) whereas females feeding on pelagic low-trophic level prey (i.e. crustaceans; Table 2); fish has a higher trophic level than crustaceans [106]. Moreover, within males, some individuals clearly fed on a higher trophic level than other males whereas within females, some individuals fed on a lower trophic level than others. This suggests that, within sexes, there are clear feeding/habitat segregations not identified before, which may be a reflection of the abnormal environmental conditions causing individuals to search for alternative prey. Individual feeding preferences and adjustments to spatial, seasonal and inter-annual variations in resources by exploiting different foraging niches were found in several other seabird species [39]. Therefore, fluctuations in individual specialization within a population may be related to temporal changes in the availability and predictability of resources [107], and could be, to some extent, a consequence of dietary sexual specializations. Indeed, when assessing sex-related specializations, females feeding on Antarctic krill (i.e. individuals that fed more than > 80% on Antarctic krill) versus males feeding on fish (i.e. individuals that fed more than > 80% on fish), females clearly feed on lower trophic levels (on pelagic crustaceans) in comparison with males (on high trophic levels (on benthic/bentho-pelagic fish); Fig 4). Therefore, in this abnormal season of low food availability, males and females do exploit different prey in a consistent way (i.e. in various foraging trips, in various days, as confirmed by the red blood cells data).

Despite the significant differences in feather carbon signatures for the end of summer (March-April; at the end of their breeding period, when their feathers were created), biologically both sexes may explore similar habitats, as these carbon stable isotopic values are similar (from Southern Ocean waters; see Results). However, at this time of the year (i.e. pre-moulting period), penguins may disperse more widely and feed in more profitable areas, despite their foraging ranges during the breeding season being still relatively short (< 50 km, and often much less) [37]. In terms of the trophic level (based on δ^15^N), no differences were found in feathers, suggesting that both males and female Gentoo penguins also fed on similar prey (likely to be *T*. *gaudichaudii*, that dominated the diet of *C*. *gunnari* [108]. Antarctic krill only represented 12% by mass in Gentoo penguins diets during their breeding period [44]).

## Supporting information

S1 Dataset Raw dataset of the diet of gentoo penguins at Bird Island, South Georgia in 2009. (XLSX)

## References

[1] Murphy EJ, Watkins JL, Trathan PN, Reid K, Meredith MP, Thorpe SE, et al Spatial and temporal operation of the Scotia Sea ecosystem: a review of large-scale links in a krill centred food web. Phil Trans R Soc B. 2007; 362: 113–148. 10.1098/rstb.2006.1957 17405210 PMC1764830

[2] Murphy EJ and Hofmann EE. End-to-end in Southern Ocean ecosystems. Current opinion in Environmental Sustainability 2012; 4: 264–271.

[3] King JC. Recent climate variability in the vicinity of the Antarctic Peninsula. Int J Climatol. 1994; 14: 357–369.

[4] Meredith MP, King JC. Rapid climate change in the ocean west of the Antarctic Peninsula during the second half of the 20^th^ century. Geophys Res Lett. 2005; 32: L19604.

[5] Kennicutt MC II, Chown SL, Cassano JJ, Liggett D, Massom R, Peck LS, et al Six priorities for Antarctic Science (and supplementary material). Nature. 2014; 512: 23–25. 10.1038/512023a 25100467

[6] Constable AJ, Melbourne-Thomas J, Corney SP, Arrigo K, Barbraud C, Barnes D, et al Change in Southern Ocean ecosystems I: How changes in physical habitats directly affect marine biota. Global change biol. 2014; 20: 3004–3025. 10.1111/gcb.12623 24802817

[7] Gutt J, Bertler N, Bracegirdle TJ, Buschmann A, Hosie G, Isla E, et al The Southern Ocean ecosystem under multiple climate change stresses—an integrated circumpolar assessment. Global Change Biol. 2015; 21: 1434–1453. 10.1111/gcb.12794 25369312

[8] Montes-Hugo M, Doney SC, Ducklow HW, Fraser W, Martinson D, Stammerjohn SE, et al Recent changes in phytoplankton communities associated with rapid regional climate change along the Western Antarctic Peninsula. Science 2009; 323: 1470–1473. 10.1126/science.1164533 19286554

[9] Reid K, Croxall JP. Environmental response of upper trophic-level predators reveals a system change in an Antarctic marine ecosystem. Proc R Soc B. 2001; 268: 377–384. 10.1098/rspb.2000.1371 11270434 PMC1088617

[10] Ducklow HW, Baker K, Martinson DG, Quetin LB, Ross RM, Smith RC, et al Marine pelagic ecosystems: the West Antarctic Peninsula. Phil Trans R Soc Lond B. 2007; 362: 67–94. 17405208 10.1098/rstb.2006.1955 PMC1764834

[11] Trivelpiece WZ, Hinke JT, Miller AK, Reiss CS, Trivelpiece SG, Watters JM, et al Variability in krill biomass links harvesting and climate warming to penguin population changes in Antarctica. Proc Natl Acad Sci USA. 2011; 108: 7625–7628. 10.1073/pnas.1016560108 21482793 PMC3088573

[12] Croxall JP, Trathan PN, Murphy EJ. Environmental change and Antarctic Seabird populations. Science 2002; 297: 1510–1514. 10.1126/science.1071987 12202819

[13] Forcada J, Trathan PN. Penguin responses to climate change in the Southern Ocean. Global Change Biol. 2009; 15: 1618–1630.

[14] Xavier JC, Peck LS. LIfe beyond the ice: marine ecosystems in the Southern Ocean In: Liggett D., Storey B., Cook Y. and Meduna V., editors. Exploring the last continent. Springer International Publishing, Switzerland 2015; pp. 229–252.

[15] Brooke ML. The food consumption of the world´s seabirds. Proc R Soc Lond B. 2004; 271: S246–S248. 10.1098/rsbl.2003.0153 PMC1810044 15252997

[16] Croxall JP, Prince PA. Seabirds as predators on marine resources, especially krill, at South Georgia In: Croxall J. P., editor editors. Seabirds: Feeding Ecology and Role in Marine Ecosystems. Cambridge: Cambridge University Press 1987; pp. 347–368.

[17] Hill SL, Keeble K, Atkinson A, Murphy EJ. A food web model to explore uncertainties in the South Georgia shelf pelagic ecosystem. Deep-Sea Res II. 2012; 59–60: 237–252.

[18] Croxall J, Williams T. The gentoo penguin as a candidate species for the CCAMLR Ecosystem Monitoring Program Scientific Committee for the Conservation of Antarctic Living Resources, Selected Scientific Papers 1991; WG-CEMP-90/14: 483–488.

[19] Xavier JC, Hill SL, Belchier M, Bracegirdle TJ, Murphy EJ, Lopes-Dias J. From ice to penguins: the role of mathematics in Antarctic research In: Bourguignon J. P., Jeltsch R., Pinto A. and Viana M., editors. Mathematics of Energy and Climate Change. CIM Series in Mathematical Sciences 2. Springer-Verlag, Switzerland 2015; pp. 389–414.

[20] Croxall JP, Reid K, Prince PA. Diet, provisioning and productivity responses of marine predators to differences in availability of Antarctic krill. Mar Ecol Prog Ser. 1999; 177: 115–131.

[21] Lescroël A, Ridoux V, Bost CA. Spatial and temporal variation in the diet of gentoo penguin (*Pygoscelis papua*) at Kerguelen Islands. Polar Biol. 2004; 27: 206–216.

[22] Lynch HJ, Naveen R, Trathan PN, Fagan WF. Spatially integrated assessment reveals widespread changes in penguin populations on the Antarctic Peninsula. Ecology 2012; 93: 1367–1377. 22834377 10.1890/11-1588.1

[23] Cimino MA, Moline MA, Fraser WR, Patterson-Fraser DL, Oliver MJ. Climate-driven sympatry may not lead to foraging competition between congeneric top-predators. Scientific reports 2016; 6: 18820 10.1038/srep18820 26732496 PMC4702144

[24] Croxall JP, Davis LS. Penguins: paradoxes and patterns. Marine Ornithology 1999; 27: 1–12.

[25] Tanton JL, Reid K, Croxall JP and Trathan PN Winter distribution and behaviour of gentoo penguins *Pygoscelis papua* at South Georgia. Polar Biol. 2004; 27: 299–303.

[26] Williams TD, Briggs DR, Croxall JP, Naito Y, Kato A. Diving pattern and performance in relation to foraging ecology in the gentoo penguin *Pygoscelis papua*. J Zool. 1992; 227: 211–230.

[27] Kato A, Williams TD, Barton TR, Rodwell S. Short-term variation in the winter diet of gentoo penguins *Pygoscelis papua* at South Georgia during July 1989. Marine Ornithology 1991; 19: 31–38.

[28] Williams TD. Foraging ecology and diet of gentoo penguins *Pygoscelis papua* at South Georgia during winter and an assessment of their winter krill consumption. Ibis. 1991; 133: 3–13.

[29] Renner M, Valencia J, Davis LS, Saez D, Cifuentes O. Sexing of adult Gentoo Penguins in Antarctica using morphometrics. Colonial Waterbirds 1998: 444–449.

[30] Bost CA, Jouventin P. Evolutionary ecology of the Gentoo penguin *Pygoscelis papua* In: Davis L. S. and Darby J., editors. Penguins. Ac. Press Orlando, Florida, USA 1990; pp. 85–112.

[31] Bost CA, Jouventin P, Pincson du Sel N. Morphometric variability on a microgeographical scale in two inshore seabirds. J Zool. 1992; 226: 135–149.

[32] Lewis S, Benvenuti S, Dall'Antonia L, Griffiths R, Money L, Sherratt TN, et al Sex-specific foraging behaviour in a monomorphic seabird. Proc Roy Soc Lond B. 2002; 269: 1687–1693. 10.1098/rspb.2002.2083 PMC1691079 12204129

[33] Gray CM, Hamer KC. Food-provisioning behaviour of male and female Manx shearwaters, *Puffinus puffinus*. Animal Behav. 2001; 62: 117e121.

[34] Quillfeldt P, Masello JF, Hamer KC. Sex differences in provisioning rules and honest signalling of need in Manx shearwaters, *Puffinus puffinus*. Animal Behav. 2004; 68: 613e620.

[35] Wearmouth VJ, Sims DW. Sexual segregation in marine fish, reptiles, birds and mammals: behaviour patterns, mechanisms and conservation implications. Adv mar biol. 2008; 54: 107–170. 10.1016/S0065-2881(08)00002-3 18929064

[36] Ruckstuhl K, Neuhaus P. Sexual segregation in vertebrates. Cambridge University Press 2005;

[37] Ratcliffe N, Trathan PN A review of the diet and at sea-distribution of penguins breeding within the CCAMLR convention area. CCAMLR Science. 2011; 18: 75–114.

[38] Cherel Y, Hobson KA, Guinet C, Vanpe C. Stable isotopes document seasonal changes in trophic niches and winter foraging individuals specialization in diving predators from the Southern Ocean. J Anim Ecol. 2007; 76: 826–836. 10.1111/j.1365-2656.2007.01238.x 17584388

[39] Ceia FR, Ramos JA. Individual specialization in the foraging and feeding strategies of seabirds: a review. Mar Biol. 2015; 162: 1923–1938.

[40] Carravieri A, Bustamante P, Churlaud C, Cherel Y. Penguins as bioindicators of mercury contamination in the Southern Ocean: Birds from the Kerguelen Islands as a case study. Science of the Total Environ. 2013; 454: 141–148. 10.1016/j.scitotenv.2013.02.060 23542487

[41] Bolnick DI, Smith T, Can intraspecific competition drive disruptive selection? An experimental test in natural populations of sticklebacks. Evolution. 2004; 58: 608–618. 15119444

[42] Ropert-Coudert Y, Kato A, Meyer X, Pellé M, MacIntosh AJJ, et al A complete breeding failure in an Adélie penguin clony correlates with unusual and extreme environmental events. Ecography. 2015; 38: 111–113.

[43] Whitehouse MJ, Meredith MP, Rothery P, Atkinson A, Ward P, Korb R. Rapid warming of the ocean around South Georgia, Southern Ocean, during the 20th Century: Forcings, characteristics and implications for lower trophic levels. Deep-Sea Research I. 2008; 55: 1218–1228.

[44] Hill S, Belchier M, Collins MA, Fielding S, Murphy EJ, Trathan PN. Multiple indicators suggest a strong ecosystem anomaly at South Georgia in 2009. WG-EMM-09/23.

[45] Fielding S, Watkins JL, Trathan PN, Enderlein P, Waluda CM, Stowasser G. Interannual variability in Antarctic krill (*Euphausia superba*) density at South Georgia, Southern Ocean: 1997–2013. ICES J Mar Sci. 2014; 71: 2578–2588.

[46] Fielding S, Watkins JL, Collins MA, Enderlein P, Venables HJ. Acoustic determination of the distribution of fish and krill across the Scotia Sea in Spring 2006, summer 2008 and autumn 2009. Deep-Sea Research II. 2012; 59–60: 173–188.

[47] Schreiber EA. Climate and weather effects on seabirds In: Schreiber E. A. and Burger J., editors. Biology of Marine Birds. CRC Press, Boca Raton, Florida 2002; pp. 179e216.

[48] Ainley DG, Wilson PR, Barton KJ, Ballard G, Nur N, Karl B. Diet and foraging effort of Adélie penguins in relation to pack-ice conditions in the southern Ross Sea. Polar Biol. 1998; 20: 311–319.

[49] Baduini C, Hyrenbach K, Coyle K, Pinchuk A, Mendenhall V, Hunt G. Mass mortality of short‐tailed shearwaters in the south‐eastern Bering Sea during summer 1997. Fisheries Oceanogr. 2001; 10: 117–130.

[50] Harris MP, Wanless S. Differential responses of Guillemot *Uria aalge* and Shag *Phalacrocorax aristotelis* to a late winter wreck. Bird Study 1996; 43: 220–230.

[51] Tranquilla LM, Hedd A, Burke C, Montevecchi WA, Regular PM, Robertson GJ, et al High Arctic sea ice conditions influence marine birds wintering in Low Arctic regions. Estuarine, Coastal and Shelf Sci. 2010; 89: 97–106.

[52] Fisher J, Lockley RM. Sea Birds. Collins, London. 1954;

[53] Lewis S, Sherratt TN, Hamer KC, Wanless S. Evidence of intra-specific competition for food in a pelagic seabird. Nature. 2001; 412: 816–819. 10.1038/35090566 11518965

[54] Selander RK. Sexual selection and dimorphism in birds In: Champbell B., editor editors. Sexual selection and the descent of Man. Heinemann, Chicago 1972; pp. 180–230.

[55] Mancini PL, Bond AL, Hobson KA, Duarte LS, Bugoni L. Foraging segregation in tropical and polar seabirds: Testing the Intersexual Competition Hypothesis. J Exp Mar Biol and Ecol. 2013; 449: 186–193.

[56] Ceia FR, Phillips RA, Ramos JA, Cherel Y, Vieira RP, Richard P, et al Short- and long-term consistency in the foraging niche of wandering albatrosses. Mar Biol. 2012; 159: 1581–1591.

[57] Xavier JC, Trathan PN, Croxall JP, Wood AG, Podestá GP, Rodhouse PG. Foraging ecology and interactions with fisheries of wandering albatrosses at South Georgia. Fisheries Oceanogr. 2004; 13: 324–344.

[58] Xavier JC, Croxall JP, Reid K. Inter-annual variation in the diet of two albatross species breeding at South Georgia: implications for breeding performance. Ibis. 2003; 145: 593–610.

[59] Kane JE. The distribution of *Parathemisto gaudichaudii* (Guer.), with observations on its life-history in the 0° to 20° E sector in the Southern Ocean. Discov Rep. 1966 34: 163–198.

[60] Boltovskoy D. South Atlantic zooplankton. Netherlands: Backhuys Publishers, Leiden 1999; 1–1706 p.

[61] Hecht T A. guide to the otoliths of Southern Ocean fishes. South African J. of Antarctic Res. 1987; 17: 2–87.

[62] Williams R, McEldowney A. A guide to the fish otoliths from waters off the Australian Antarctic Territory, Heard and Macquarie Island. ANARE Research Notes. 1990; 173 p.

[63] Smale MJ, Watson G, Hecht T. Otolith atlas of Southern African marine fishes. Ichthyological Monographs of the JLB Smith Institute of Ichthyology. Grahamstown, South Africa. 1995; 1–253 p.

[64] Reid K. A guide to the use of otoliths in the study of predators at South Georgia. Cambridge: British Antarctic Survey 1996; 40 p.

[65] Adams NJ, Klages NT. Seasonal variation in the diet of king penguin *Aptenodytes patagonicus* at Sub-Antarctic Marion Island. J Zool. 1987; 212: 303–324.

[66] Olsson O, North AW. Diet of the king penguin *Aptenodytes patagonicus* during three summers at South Georgia. Ibis. 1997; 139: 504–512.

[67] Berrow SD, Croxall JP. The diet of white-chinned petrels *Procellaria aequinoctialis*, Linnaeus 1758, in years of contrasting prey availability at South Georgia. Antarctic Sci. 1999; 11: 283–292.

[68] Croxall JP, North AW, Prince PA. Fish prey of the wandering albatross *Diomedea exulans* at South Georgia. Polar Biol. 1988; 9: 9–16.

[69] Croxall JP, Hall AJ, Hill HJ, North AW, Rodhouse PG. The food and feeding ecology of the white-chinned petrel *Procellaria aequinoctialis* at South-Georgia. J Zool. 1995; 237: 133–150.

[70] Xavier JC, Cherel Y. Cephalopod beak guide for the Southern Ocean. British Antarctic Survey 2009; 129 p.

[71] Newsome SD, Rio CMD, Bearhop S, Phillips DL. A niche for isotopic ecology. Front Ecol Environ. 2007; 5: 429–436.

[72] Cherel Y, Connan M, Jaeger A, Richard P. Seabird year-round and historical feeding ecology: blood and feather δ13C and δ15N values document foraging plasticity of small sympatric petrels. Mar Ecol Prog Ser. 2014; 505: 267–280.

[73] Hobson KA, Clark RG. Turnover of δ13C in cellular and plasma reactions of blood: implications for nondestructive sampling in avian dietary studies. Auk 1993; 110: 638–641.

[74] Votier SC, Bearhop S, Witt MJ, Inger R, Thompson D, Newton J. Individual responses of seabirds to commercial fisheries revealed using GPS tracking, stable isotopes and vessel monitoring systems. J Appl Ecol. 2010 47: 487–497.

[75] Davis RW, Croxall JP, O'Connell MJ. The reproductive energetics of gentoo (*Pygoscelis papua*) and macaroni (*Eudyptes chrysolophus*) penguins at South Georgia. (SC-CAMLR-VIII/BG/14) 1989; 58: 59–74.

[76] Cherel Y, Hobson KA, Weimerskirch H. Using stable isotopes to study resource acquisition and allocation in procellariiform seabirds. Oecologia 2005; 145: 533–540. 10.1007/s00442-005-0156-7 16001219

[77] Cherel Y, Hobson KA, Hassani S. Isotopic discrimination between food and blood and feathers of captive penguins: implications for dietary studies in the wild. Physiol and Biochem Zool. 2005; 78: 106–115. 15702469 10.1086/425202

[78] Jackson AL, Inger R, Parnell AC, Bearhop S. Comparing isotopic niche widths among and within communities: SIBER—Stable Isotope Bayesian Ellipses in R. J Anim Ecol. 2011; 80: 595−602. 10.1111/j.1365-2656.2011.01806.x 21401589

[79] Layman CA, Arrington DA, Montaña CG, Post DM. Can stable isotope ratios provide for community-wide measures of trophic structure? Ecology. 2007; 88: 42–48. 17489452 10.1890/0012-9658(2007)88[42:csirpf]2.0.co;2

[80] Parnell AC, Inger R, Bearhop S, Jackson AL. Source partitioning using stable isotopes: coping with too much variation. PLoS ONE. 2010; 5: e9672 10.1371/journal.pone.0009672 20300637 PMC2837382

[81] Walsh PS, Metzger DA, Higuchi R. Chelex-100 as a medium for simple extraction of DNA for PCR-based typing from forensic material. Biotechniques. 1991; 10: 506–513. 1867860

[82] Griffiths R, Double MC, Orr K, Dawson RJG. A DNA test to sex most birds. Molecular Ecol. 1998; 7: 1071–1075. 10.1046/j.1365-294x.1998.00389.x 9711866

[83] Bertellotti M, Tella JL, Godoy JA, Blanco G, Forero MG, Donázar JA, et al Determining sex of Magellanic penguins using molecular procedures and discriminant functions. Waterbirds. 2002 25(4): 479–484.

[84] Constantini V, Guaricci AC, Laricchiuta P, Rausa P, Lacalandra GM. DNA sexing in Humboldt Penguins (*Spheniscus humboldti*) from feather samples. Animal Reproduction Sci. 2008 106: 162–167. 10.1016/j.anireprosci.2007.12.013 18258392

[85] Williams TD. Annual variation in breeding biology of gentoo penguins, (Pygoscelis papua) at Bird Island, South Georgia. (WG-CEMP-90/38) 1990.

[86] R Core Team. R: a language and environment for statistical computing. R Foundation for Statistical Computing, Viena. 2013;

[87] Berrow SD, Taylor RI, Murray AWA. Influence of sampling protocol on diet determination of gentoo penguins *Pygoscelis papua* and Antarctic fur seals *Arctocephalus gazella*. Polar Biol. 1999; 22: 156–163.

[88] Brierley AS, Watkins JL, Goss C, Wilkinson MT, Everson I. Acoustic estimates of krill density at South Georgia, 1981 to 1998. CCAMLR Science. 1999; 6: 47–57.

[89] Croxall JP, Davis RW, Oconnell MJ. Diving patterns in relation to diet of Gentoo and Macaroni Penguins at South Georgia. Condor. 1988; 90: 157–167.

[90] Croxall JP, Prince PA. The food of gentoo penguins *Pygoscelis papua* and macaroni penguins *Eudyptes chrysolophus* at South Georgia. Ibis. 1980; 122: 245–253.

[91] Bearhop S, Phillips RA, McGill R, Cherel Y, Dawson DA, Croxall JP. Stable isotopes indicate sex-specific and long-term individual foraging specialization in diving seabirds. Mar Ecol Prog Ser. 2006; 311: 157–164.

[92] Forero MG, Hobson KA, Bortolotti GR, Donázar JA, Bertellotti M, Blanco G. Food resource utilisation by Magellanic penguin evaluated through stable isotope analysis: segregation by sex and age and influence of offspring quality. Mar Ecol Prog Ser. 2002; 234: 289–299.

[93] Ludynia K, Dehnhard N, Poisbleau M, Demongin L, Masello JF, Voigt CC, et al Sexual segregation in rockhopper penguins during incubation. Animal Behav. 2013; 85: 255–267.

[94] Rey AR, Pütz K, Scioscia G, Lüthi B, Schiavini A. Sexual differences in the foraging behaviour of Magellanic Penguins related to stage of breeding. Emu. 2012; 112: 90–96.

[95] Green JA, Boyd IL, Woakes AJ, Warren NL, Butler PJ. Behavioural plasticity during year-round foraging in Macaroni Penguins. Mar Ecol Prog Ser. 2005; 296: 183–196.

[96] Gon O, Heemstra PC. Fishes of the Southern Ocean. Grahamstown, South Africa: JLB Smith Institute of Ichthyology 1990;

[97] McKenna JEJ. Trophic relationships within the Antarctic demersal fish community of South Georgia Island. Fishery Bull. 1991; 89: 643–654.

[98] Bost C-A, Lage J, Putz K. Maximum diving depth and diving patterns of the Gentoo penguin *Pygoscelis papua* at the Crozet Islands. Marine Ornit. 1994; 22: 237–244.

[99] Volkman NJ, Jazdzewski K, Kittel W, Trivelpiece WZ. Diets of *Pygoscelis* Penguins at King George Island, Antarctica. Condor. 1980; 82: 373–378.

[100] Chappell MA, Janes DN, Shoemaker VH, Bucher TL, Maloney SK. Reproductive effort in Adélie penguins. Behav Ecol Sociobiol. 1993; 33: 173–182.

[101] Clarke J, Manly B, Kerry K, Gardner H, Franchi E, Corsolini S., et al Sex differences in Adélie penguin foraging strategies. Polar Biol. 1998; 20: 248–258.

[102] Dehnhard N, Voigt CC, Poisbleau M, Demongin L, Quillfeldt P. Stable isotopes in southern rockhopper penguins: foraging areas and sexual differences in the non-breeding period. Polar Biol. 2011; 34: 1763–1773.

[103] Masello JF, Mundry R, Poisbleau M, Demongin L, Voigt CC, Wikelski M., et al Diving seabirds share foraging space and time within and among species. Ecosphere. 2010; 1: 1–28.

[104] Wienecke BC, Robertson G. Foraging space of emperor penguins *Aptenodytes forsteri* in Antarctic shelf waters in winter. Mar Ecol Prog Ser. 1997; 159: 249–263.

[105] Taylor SS, Leonard ML, Boness DJ, Majluf P. Foraging by Humboldt penguins (*Spheniscus humboldti*) during the chick-rearing period: general patterns, sex differences, and recommendations to reduce incidental catches in fishing nets. Canadian J. Zool. 2002; 80: 700–707.

[106] Stowasser G, Atkinson A, McGill RAR, Phillips RA, Collins MA, Pond DW. Food web dynamics in the Scotia Sea in summer: A stable isotope study. Deep Sea Research Part II. 2012; 59–60: 208–221.

[107] Ceia FR, Paiva VH, Garthe S, Marques JC, Ramos JA. Can variations in the spatial distribution at sea and isotopic niche width be associated with consistency in the isotopic niche of a pelagic seabird species? Marine Biol. 2014; 161: 1861–1872.

[108] Main CE, Collins MA, Mitchell R, Belchier M. Identifying patterns in the diet of mackerel icefish (*Champsocephalus gunnari*) at South Georgia using bootstrapped confidence intervals of a dietary index. Polar Biol. 2009; 32: 569–581.

[109] Reynolds RW, Rayner NA, Smith TM, Stokes DC, Wang W. An Improved In Situ and Satellite SST Analysis for Climate. J Climate. 2002; 15: 1609–1625.

[110] Angelier F, Bost CA, Giraudeau M, Bouteloup G, Dano S, Chastel O. Corticosterone and foraging behavior in a diving seabird: The Adélie penguin, *Pygoscelis adeliae*. Gen Comp Endocrinol. 2008; 156: 134–144. 10.1016/j.ygcen.2007.12.001 18221738

[111] Watanuki Y, Takahashi A, Sato K. Feeding area specialization of chick-rearing Adélie penguins *Pygoscelis adeliae* in a fast sea-ice area. Ibis. 2003; 145: 558–564.

[112] Gorman KB, Williams TD, Fraser WR. Ecological sexual dimorphism and environmental variability within a community of Antarctic Penguins (Genus *Pygoscelis*). PloSONE. 2014; 9: e90081. 10.1371/journal.pone.0090081 PMC3943793 24599330

[113] Trivelpiece WZ, Trivelpiece SG, Volkman NJ, Ware SH. Breeding and feeding ecologies of Pygoscelid penguins. Ant J US. 1983; 18: 209–210.

[114] Baylis AMM, Orben RA, Pistorius P, Brickle P, Staniland I, Ratcliffe N. Winter foraging site fidelity of king penguins breeding at the Falkland Islands. Mar Biol. 2015; 162: 99–110.

[115] Bethge P, Nicol S, Culik B, Wilson R. Diving behaviour and energetics in breeding little penguins (*Eudyptula minor*). J Zool. 1997; 242: 483–502.

[116] Chiaradia A, Forero MG, Hobson KA, Swearer SE, Hume F, Renwick L, et al Diet segregation between two colonies of little penguins *Eudyptula minor* in southeast Australia. Austral Ecol. 2012; 37: 610–619.

[117] Ropert-Coudert Y, Kato A, Naito Y, Cannell B. Individual diving strategies in the little penguin. Waterbirds. 2003; 4: 403–408.

[118] Barlow KE, Croxall JP. Seasonal and interannual variation in foraging range and habitat of macaroni penguins at South Georgia. Mar Ecol Prog Ser. 2002; 232: 291–304.

[119] Hull CL. Comparative diving behaviour and segregation of the marine habitat by breeding royal penguins, *Eudyptes schlegeli*, and eastern rockhopper penguins, *Eudyptes chrysocome filholi*, at Macquarie Island. Can J Zool. 2000; 78: 333–345.

[120] Seddon P, van Heezik Y. Diving depths of the yellow-eyed penguin *Megadyptes antipodes*. Emu. 1990; 90: 53–57.

